# Effects of a Daily Mile Program During Recess on Physical Fitness in Adolescents: A Comparative Pilot Study of Weekly Frequency and Gender Differences Among Students in a Region of Spain

**DOI:** 10.3390/sports13070217

**Published:** 2025-07-07

**Authors:** Rubén Navarro-Patón, Miguel Cons-Ferreiro, María Muíño-Piñeiro, Marcos Mecías-Calvo

**Affiliations:** 1 Facultade de Formación do Profesorado (Campus Terra), Universidade de Santiago de Compostela, 27001 Lugo, Spain; miguel.cons@usc.es (M.C.-F.); mariamuino.pineiro@usc.es (M.M.-P.); marcos.mecias@usc.es (M.M.-C.); 2 Research Group on Motor Skills, Physical Education, and Health, Universidade de Santiago de Compostela, 27001 Lugo, Spain

**Keywords:** physical activity, secondary education, endurance, strength, speed, flexibility

## Abstract

Background: Recess provides a valuable opportunity for physical activity for students at school. However, there is no clear evidence regarding the effects of running a mile daily on schoolchildren’s physical fitness. The objective of this study was to identify and evaluate the effects of running a mile daily during recess in a school setting over a 12-week period. Methods: The study included 68 students (39 boys and 29 girls) aged 13 to 16 years (14.45 ± 1.08) from secondary education (Spain). Participants were randomly assigned to one of three groups. Intervention Group 1 (IG1): one-mile run once per week (22 students). Intervention Group 2 (IG2): one-mile runs three times per week (21 students). Intervention Group 3 (IG3): one-mile runs five times per week (25 students). Physical fitness was assessed using the following tests: Broad jump (lower limb strength); Sit and Reach (lower limb flexibility); 4 × 10 m shuttle runs (speed, agility, and coordination); 10 × 5 m shuttle runs (displacement speed); 20 m shuttle run (Course Navette) (cardiorespiratory endurance). Results: Broad jump: No overall post-intervention differences were observed (p > 0.05), but there were pre-existing gender differences [boys outperform girls in IG2 and IG3 (p < 0.05)] that widened after the program, even appearing in IG1 (p = 0.031). Sit and Reach: No overall changes occurred (p > 0.05), but gender differences emerged in IG3 after the intervention [girls outperformed boys (p < 0.050)], and IG3 boys showed a decrease in flexibility after the program (p = 0.041). The 4 × 10 m shuttle runs: Initial differences between IG1 vs. IG3 disappeared after the intervention, with an overall increase in test time (p > 0.005). Pre-existing gender differences decreased, except in IG2 (boys remained faster; p < 0.001). The 10 × 5 m shuttle runs: Significant improvements were observed in all post-intervention groups (p = 0.003), with the greatest gains in IG3 boys (p < 0.001) and IG1 girls (p = 0.003). The 20 m shuttle run: Significant improvements occurred in IG1 and IG3 (p < 0.005), particularly in IG3 boys (p = 0.002) and IG1 girls (p = 0.019). Conclusions: Although effects varied by fitness component, intervention frequency, and gender, daily mile running was shown to be a viable strategy for improving aspects of fitness in adolescents, particularly endurance and speed, even at lower frequencies.

## 1. Introduction

Physical activity provides health benefits such as physical fitness, mental well-being, and long-term disease prevention, which have been reported and documented (World Health Organization [WHO], 2020) [1]. Adolescence is an appropriate stage to establish lifelong healthy habits, as regular physical activity during this stage improves cardiovascular health, muscle strength, and metabolic function [2]. Conversely, physical inactivity is linked to obesity, insulin resistance, and poor mental health, among others [3]. Despite these benefits, global levels of physical inactivity among adolescents remain alarmingly high, with only 20% of young people achieving the 60 min of moderate-to-vigorous physical activity (MVPA) recommended by the WHO [4].

Physical fitness is a multidimensional concept encompassing aerobic endurance, muscular strength, speed, and flexibility [5]. Gender differences in physical fitness during adolescence are well established, with boys typically outperforming girls in aerobic capacity and strength due to physiological and hormonal factors [6]. However, the extent to which structured physical activity interventions can mitigate these differences remains to be studied. For example, scientific evidence indicates that improvements in aerobic endurance resulting from running programs may be more pronounced in boys, whereas girls may benefit more from flexibility and coordination exercises [7,8]. Therefore, understanding these nuances is necessary to design interventions tailored to the entire school population.

Schools, as settings where young people spend an average of 5 h daily, represent an ideal environment for promoting physical activity interventions [9] and an opportunity to integrate structured physical activity interventions, especially during recess [10]. This recess has traditionally been an underutilized period for systematic exercise [11] but offers a daily opportunity for structured and unstructured physical activity [12]. However, its potential is often underestimated in favor of academic priorities [10,13]. Structured recess interventions, such as The Daily Mile program, may offer an inclusive, cost-effective, and time-efficient method for increasing moderate-to-vigorous physical activity (MVPA) [14]. Among these interventions, The Daily Mile program, which involves running or walking one mile (1.6 km) during school recess, has gained traction as a simple and affordable approach to increasing daily physical activity in schoolchildren [15]. A recent study by Dring et al. [16] showed that 5 weeks of participation in The Daily Mile significantly improved cardiorespiratory fitness in children, although no significant effects were found on cognitive function or body composition.

Preliminary studies suggest that these interventions improve cardiovascular fitness, body composition, and psychological well-being [15,17]. However, most research has focused on young children, with limited data on adolescents, a population experiencing distinctive physiological and behavioral changes that may influence intervention outcomes [18]. Furthermore, the optimal frequency of the daily mile remains unclear. While daily participation may maximize benefits, logistical constraints in schools necessitate exploring whether less frequent implementation (e.g., 1–3 days per week) produces comparable improvements. Despite this, scientific evidence on its effectiveness remains limited, particularly regarding optimal frequency and gender adaptations.

Therefore, the objectives of this study were (1) to evaluate the effects of a 12-week Daily Mile program on physical fitness (lower body strength and flexibility; displacement speed; speed, agility, and coordination; and cardiorespiratory endurance) in adolescents; (2) to compare outcomes across three intervention frequencies (1, 3, and 5 days per week); and (3) to examine gender differences in fitness adaptations.

## 2. Materials and Methods

### 2.1. Study Design

This study follows a quasi-experimental design [19], with a controlled intervention (The Daily Mile program) applied to three groups of participants (IG1, IG2, and IG3) with different weekly frequencies. Group assignment was non-random. Pre- and post-intervention measurements were performed to assess changes in physical variables (strength, flexibility, speed, agility, and cardiorespiratory endurance), also considering gender differences, focusing on within-group comparisons and analyses (before–after in each group), between-group comparisons (IG1 vs. IG2 vs. IG3), and the effects according to the gender of the participants.

The Ethics Committee of the national platform EDUCA approved the research with the code number 08/2024, approved on 10 July 2024. Participants received an informed consent form and written approval; parents/legal guardians signed the informed consent form, and adolescents gave verbal assent to participate. They were informed that their participation was voluntary and that they could withdraw from the study at any time. During their participation in this study, subjects were treated in accordance with the Declaration of Helsinki.

### 2.2. Participants

The study population consisted of compulsory secondary education students aged 13 to 16 from the same school. They were divided into three intervention groups, selected by convenience to facilitate the study. The first intervention group (IG1; n = 37) consisted of students who ran a mile once a week during school recess. The second intervention group (IG2; n = 37) consisted of students with similar characteristics to IG1, but who ran a mile three days a week. Intervention Group 3 (IG3; n = 36) consisted of students with similar characteristics to IG1 and IG2 but who ran a mile five days a week.

All high school students were invited to participate. The following inclusion/exclusion criteria were considered for participation in the study (e.g., health problems, refusal of consent, absenteeism): Students were excluded if they did not provide signed informed consent from their parent or legal guardian [i.e., IG1 (n = 5); IG2 (n = 6); IG3 (n = 4)] were excluded; those who did not complete 80% of the daily mileage sessions due to illness or injury [i.e., IG1 (n = 6); IG2 (n = 6); IG3 (n = 3)]; and those who were not present at the initial or final data collection [i.e., IG1 (n = 4); IG2 (n = 4); IG3 (n = 4)].

### 2.3. Tools and Measurements

First, sociodemographic data were collected from the participants: age and gender (men/women/other). Next, the weight and height of all participants, who were dressed in comfortable clothing, were measured. Based on their weight and height, the body mass index (BMI) was calculated using the formula [weight (kg)/height (m^2^)]. This was achieved using a scale and a height rod.

The room used for this part of the study had a comfortable temperature and allowed for privacy for the participants. Measurements were performed according to standard protocols and uniformly across all three intervention groups by the same researchers and a school representative.

#### 2.3.1. Collecting Data from Physical Tests

For data collection, some tests from the DAFIS Battery of the Xunta de Galicia (Galician Regional Government), with competencies in education, were used. This battery includes the elements described below (https://dafis.xunta.es/index.php accessed on 3 May 2025), and one more was added (10 × 5 m). Participants were informed that they should not perform any intense physical activity at least 48 h before the tests. Participants performed a 5 min warm-up before starting the tests. The tests were carried out during the same week in physical education classes (two 50 min sessions) with the following sequence: Day 1: Weight and height (BMI); flexibility; broad jump and 4 × 10 m speed-agility test; Day 2: Speed test (10 × 5 m) and 20 m shuttle test. The exercises are described as follows:

***a.***  ***Broad Jump:*** The objective of this test is to assess the explosive strength of the lower extremities. To perform it, the participant stands behind a line with their feet shoulder-width apart. From this position, they bend their knees and push off with intensity and simultaneously with both legs, trying to reach the maximum distance. Swinging the arms is permitted. The participant must land simultaneously with both feet and remain standing. The test is repeated twice. The score is obtained from the distance between the takeoff line and the point where the back of the heel landed closest to the starting line. The distance is measured in centimeters. A repeat jump is permitted if the participant loses balance during the attempt.
***b.***  ***Sit and Reach:*** The purpose of this test is to assess the range of motion of trunk and hamstring flexion. The measurement is performed with the participant barefoot, seated with both legs extended and feet resting on the box or testing apparatus. Then, with palms facing down, the student leans forward with both hands along the scale four times and holds the position on the fourth attempt for at least one second. The distance achieved is measured. Flexibility is measured in centimeters.
***c.***  ***The 4 × 10 m run:*** The purpose of this test is to comprehensively assess movement speed, agility, and coordination. The student will complete four 10 m shuttle runs. In the first run, the student will travel without a sponge at top speed from the starting line to the finish line, located 10 m away. There, they will pick up a sponge (A) and return at top speed to the starting point, where they will leave the moved sponge (A) and pick up a second sponge (B). They will make a third move to deposit the sponge and pick up a final sponge (C), which they will use to travel at top speed to the starting line. The time taken to complete this circuit is measured in seconds. Two attempts are allowed, and the best attempt will be recorded.
***d.***  ***The 10 × 5 m run:*** The purpose of this test is to assess the participant’s displacement speed. It consists of running 5 m 10 times, i.e., 5 times the round trip. You will run to the 5 m line, step onto it, and return to the starting line (this 5 times). Upon reaching the finish line, you will cross the line, not just step onto it, and the timer will stop. The time will be recorded in seconds. Two attempts are allowed, and the best will be recorded.
***e.***  ***Course Navette:*** The purpose of this test is to assess the participant’s cardiorespiratory fitness level. A 20 m shuttle run. The starting speed is 8.5 km/h, increasing by 0.5 km/h per minute (1 min equals one period). Students should be instructed to run in a straight line, from one pivot to the next, keeping time to the audible signal, so that they reach the end of each 20 m distance at the time the audible signal is given, within 1 or 2 m (1 or 2 m). At the end of each 20 m run, they must touch the line with their foot, turn around, and continue in the other direction. The test ends when the student cannot complete two runs simultaneously to the audible signals. The last completed period is recorded.

#### 2.3.2. Organized Physical Activity Program (Daily Mile)

The school-based organized physical activity program was implemented for 12 weeks during the second 30 min break in the morning (i.e., mid-morning from 11:50 a.m. to 12:20 p.m.). The daily mile was completed on a circuit around the school’s outdoor area, which combined indoor and outdoor areas. Participants were required to complete five laps of a 322 m circuit, measured by GPS and marked with posts so that students had to do it from the outside. IG1 participants were required to complete this intervention program one day a week; IG2 participants were required to complete it three days a week; and IG3 participants were required to complete it five days a week. Students could walk or run the circuit, depending on their ability and experience. Before starting the daily mile, students performed a five-minute warm-up consisting of (1) a General Pulse Raiser (2 min) (ACSM guidelines for youth warm-ups [20]): Light jogging or brisk walking around a designated area (e.g., playground or track). Gradual progression from walking to jogging to elevate core temperature. (2) Dynamic Stretches (2 min; [21]: Leg swings (front-to-back and side-to-side, 10 reps per leg): Mobilizes hips and hamstrings. Walking lunges (5 reps per leg): Activates quadriceps and glutes. High knees (20 s): Engages hip flexors and coordination. Butt kicks (20 s): Warms up hamstrings and knees. (3) Neuromuscular Activation (1 min; [22]): Short accelerations (2–3 bursts of 10 m runs at 50–70% effort). Side shuffles (10 s each direction): Prepares for agility demands.

### 2.4. Procedures

A high school was invited to participate in the study. This school was selected for its ease of implementation and its membership in the Active Schools program of the Xunta de Galicia (Government of Galicia). Once the school was contacted and the study approved by the administration and the physical education teacher, the parents or legal guardians of the minors were informed about the purpose of the research, as well as about all the ethical precepts governing this type of research. After obtaining signed consent and the school’s acceptance, the researchers conducted a lottery to determine which students would be part of each intervention group (i.e., IG1: 1 mile 1 day per week; IG2: 1 mile 3 days per week; IG3: 1 mile 5 days per week).

**Figure 1:**
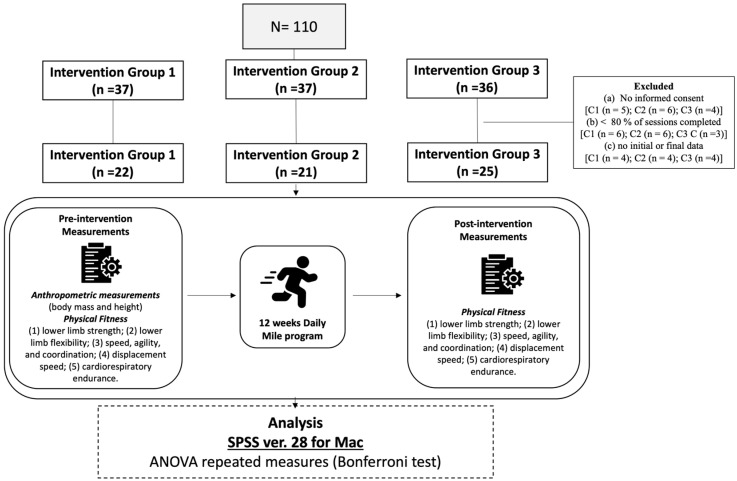
Flowchart of the research process.

Once the intervention groups were defined, physical fitness tests were administered (i.e., lower limb strength and flexibility; speed, agility, and coordination; displacement speed; and cardiorespiratory endurance). The intervention program began for 12 weeks, with each group completing one mile daily according to their intervention group protocol. Following the intervention, the students were assessed again with the same baseline tests. A flowchart of the research can be seen in Figure 1.

### 2.5. Data Analysis

SPSS (SPSS v. 28, IBM Corporation, New York, NY, USA) was used for all statistical analyses. The significance level was set at p < 0.05.

Means and standard deviations were used to express the central tendency of quantitative data, while categorical variables were expressed as frequencies and percentages. The assumptions of the three-way ANOVA were verified, confirming data normality (Kolmogorov–Smirnov test, p > 0.05). Before implementing the training programs, differences between intervention groups 1 (IG1), 2 (IG2), and 3 (IG3) in terms of age, weight, height, and BMI were assessed using a one-way ANOVA test. Pearson’s χ^2^ test was used to determine whether there were differences between intervention groups based on the gender variable. Following implementation of the 12-week Daily Mile physical activity program at the center, a three-way ANOVA [i.e., time (pre-test vs. post-test), group (IG1 vs. IG2 vs. IG3), and gender (men vs. women)] was performed, using time as the repeated measures factor. The homogeneity of variances (Levene’s test, p > 0.05) and sphericity (Mauchly’s test, p > 0.05) were verified. To analyze the possible effect of these factors on the dependent variables studied (i.e., strength and flexibility of the lower limbs; speed, agility and coordination; movement speed; and cardiorespiratory endurance), the Bonferroni statistic was used to control for type I error, ensuring the robustness of the findings, and the effect size was calculated in terms of eta squared (η^2^).

## 3. Results

A total of 68 students participated in this pilot study, divided into three cohorts: IG1 (n = 22), IG2 (n = 21), and IG3 (n = 25). Statistically significant differences were observed in BMI (p = 0.025), which was higher in IG1, but not in the other variables studied among the three intervention groups (Table 1).

### 3.1. Physical Fitness Outcomes

Table 2 shows the mean overall scores of Intervention Group 1, Intervention Group 2, and Intervention Group 3 before and after the implementation of the 12-week intervention program.

### 3.2. Lower Limb Strength Outcomes

Pre- and post-intervention results between IG1 vs. IG2 vs. IG3 indicated no statistically significant differences in overall lower limb strength (p > 0.05). No significant differences were found in the before-and-after intra-intervention group comparisons (p > 0.050). Considering the gender of the participants in this intra-intervention group comparison, significant differences were observed between girls and boys before The Daily Mile program in IG2 [F (1, 61) = 10.209, p = 0.003, η^2^ = 0.188, 95% CI: 17.392, 76.808] and in IG3 [F (1, 61) = 9.432, p = 0.030, η^2^ = 0.102, 95% CI: 4.091, 78.228], with the distance traveled by boys being greater in each cohort. Once The Daily Mile program was implemented, these differences increased in IG2 [F (1, 61) = 16.584, p < 0.001, η^2^ = 0.274, 95% CI 26.387, 78.095] and IG3 [F (1, 61) = 7.855, p = 0.008, η^2^ = 0.151, 95% CI 12.603, 77.121] and appeared in IG1 [F (1, 61) = 4.954, p = 0.031, η^2^ = 0.101, 95% CI 2.568, 51.778], with boys reaching greater distances than girls. No significant differences were found in the other comparisons (p > 0.050).

### 3.3. Lower Limb Flexibility Outcomes

Pre- and post-intervention results between IG1 vs. IG2 vs. IG3 indicated no statistically significant differences in overall lower limb flexibility (p > 0.05). No significant differences were found in the before-and-after intra-intervention group comparisons (p > 0.050). Considering the gender of the participants in this intra-intervention group comparison, before The Daily Mile program, no significant differences were observed between girls and boys in any of the intervention groups (p > 0.050). Once The Daily Mile program was implemented, statistically significant differences were observed at IG3 [F (1, 61) = 4.036, p = 0.050, η^2^ = 0.071, 95% CI: 0.014, 16.692], where girls showed greater flexibility than boys.

Finally, if boys are compared before and after the 12-week program, significant differences were found in IG3 [F (1, 61) = 4.408, p = 0.041, η^2^ = 0.077, 95% CI 0.118, 5.166], with flexibility decreasing once the physical activity program was completed.

### 3.4. Speed, Agility and Coordination Outcomes (4 × 10 m)

The results before the intervention, between IG1 vs. IG2 vs. IG3, indicated that there were statistically significant differences in speed, agility and coordination between IG1 and IG3 [F (2, 61) = 4.408, p = 0.041, η^2^ = 0.077, 95% CI 0.118, 5.166], in favor of IG3. After the 12-week intervention, previous differences disappeared (p > 0.050), as the time taken to complete the test increased in all cohorts. If gender is taken into account in these comparisons, there were differences before the intervention between boys in IG1 and IG2 [F (2, 61) = 4.066, p = 0.044, η^2^ = 0.148, 95% CI: 0.023; 2.268] and between girls in IG1 and IG3 [F (2, 61) = 3.661, p = 0.041, η^2^ = 0.135, 95% CI: 0.094; 2.457], with children in IG1 taking longer to complete the test. However, these differences disappeared after the implementation of the program (p > 0.050).

No significant differences were found in the before-and-after intra-intervention group comparisons (p > 0.050), but a trend towards longer completion times was observed for this test. Considering the gender of the participants in this intra-intervention group comparison, significant differences were observed between girls and boys before The Daily Mile program in IG1 [F (1, 61) = 7.104, p = 0.011, η^2^ = 0.131, 95% CI: 0.285, 2.039] and IG2 [F (1, 61) = 18.822, p < 0.001, η^2^ = 0.286, 95% CI: 0.971, 2.649], with the time taken by boys in each intervention group being shorter. Following the implementation of The Daily Mile program, these differences decreased, with significant differences remaining only in IG2 [F (1, 61) = 16.584, p < 0.001, η^2^ = 0.274, 95% CI 0.106, 2.336], which reduced the differences in the time spent by boys and girls on this test. No significant differences were observed in the other comparisons (p > 0.050).

### 3.5. Displacement Speed Outcomes

Pre-intervention results between IG1, IG2, and IG3 showed no statistically significant differences in displacement speed (p > 0.050). However, after the implementation of The Daily Mile program for 12 weeks, significant differences were observed between IG1 and IG3 [F (2, 61) = 4.408, p = 0.029, η^2^ = 0.077, 95% CI: 0.151, 3.425], with children in IG3 taking the least time.

Intra-intervention group comparisons showed significant differences before and after the program in IG1 [F (1, 61) = 9.283, p = 0.003, η^2^ = 0.132, 95% CI 0.261, 1.258]; IG2 [F (1, 61) = 11.331, p = 0.001, η^2^ = 0.157, 95% CI 0.334, 1.313]; and IG3 [F (1, 61) = 11.880, p = 0.001, η^2^ = 0.163, 95% CI 0.375, 1.412], with this test being performed in less time after the 12-week intervention program.

Considering the gender of participants in this within-intervention group comparison, significant differences were observed between girls and boys before The Daily Mile program in IG1 [F (1, 61) = 5.665, p = 0.021, η^2^ = 0.085, 95% CI: 0.414, 4.785] and C2 [F (1, 61) = 4.548, p = 0.037, η^2^ = 0.089, 95% CI: 0.143, 4.455], with boys spending less time in each cohort. Once The Daily Mile program was implemented, these differences remained [i.e., IG1 (p = 0.023); IG2 (p = 0.026)].

Furthermore, significant differences were found in the pre-post comparison of the 12-week intervention program between boys in IG2 [F(1, 61) = 7.246, p = 0.009, η^2^ = 0.106, 95% CI 0.210, 1.424], boys in IG3 [F(1, 61) = 18.359, p < 0.001, η^2^ = 0.231, 95% CI 0.575, 1.581], and between girls in IG1 [F(1, 61) = 9.663, p = 0.003, η^2^ = 0.137, 95% CI 0.312, 1.437] and IG2 [F(1, 61) = 4.626, p = 0.035, η^2^ = 0.070, 95% CI: 0.058, 1.602], with the time taken to complete this speed test being shorter in all cases after the intervention program. No significant differences were found in the other comparisons (p > 0.050).

### 3.6. Cardiorespiratory Endurance Outcomes

The results of the pre- and post-intervention comparison between IG1, IG2, and IG3 showed no statistically significant differences in overall cardiorespiratory endurance (p > 0.05). However, an improvement was observed in this test in all groups, increasing the level achieved at the end of the 12-week program.

Intra-intervention group comparisons showed significant differences before and after the 12-week program in IG1 [F (1, 61) = 9.432, p = 0.004, η^2^ = 0.170, 95% CI 0.364, 1.750] and IG3 [F (1, 61) = 8.760, p = 0.005, η^2^ = 0.160, 95% CI 0.356, 1.869], reaching a higher stage after the application of the 12-week program.

If gender is taken into account in this intra-intervention group comparison, significant differences appear in boys from IG3 [F (1, 61) = 11.197, p = 0.002, η^2^ = 0.196, 95% CI 0.513, 2.063] and in girls from IG1 [F (1, 61) = 5.945, p = 0.019, η^2^ = 0.114, 95% CI 0.183, 1.911], with both reaching higher stages post-intervention.

Finally, significant differences were found when comparing boys and girls within the same intervention group in IG2, both before the intervention (F (1, 61) = 6.014, p = 0.018, η^2^ = 0.116, 95% CI 0.500, 5.0078] and after (F (1, 61) = 8.478, p = 0.006, η^2^ = 0.156, 95% CI 0.818, 4.004], with the boys reaching a higher stage in both cases.

## 4. Discussion

This study evaluated the effects of a 12-week school-based Daily Mile program on various components of physical fitness in adolescents, analyzing three intervention frequencies (1, 3, and 5 days per week) and gender differences. Overall, the results reveal heterogeneous effects based on assessed physical capacity, practice frequency, and gender [23], providing relevant information for the future design of physical activity programs in educational settings [8]. For example, no overall differences were observed in lower extremity strength, but boys improved more than girls after the intervention. In flexibility, girls who ran the mile daily 5 days a week outperformed boys, as the latter’s flexibility worsened. In speed/agility, gender differences narrowed, with improvements in both boys and girls. Displacement speed improved significantly in all groups, especially in boys (IG2 and IG3) and girls (IG1 and IG2). Finally, cardiorespiratory endurance increased in all groups, with notable improvements in boys (IG3) and girls (IG1). The ecological validity of the intervention (its feasibility and effectiveness under real-life school conditions) was high in our study, as it required minimal resources and was adapted to existing schedules (recesses). Recent studies highlight that such low-workload interventions are more likely to be adopted long-term by schools [8,24]. Furthermore, the finding that even one weekly session improved cardiorespiratory endurance has practical relevance, as it fits within the limited time and resources available in many education systems [25].

Moving to a detailed assessment of each of the physical capacities analyzed to address the three objectives of this study, which were (1) to evaluate the effects of a 12-week Daily Mile program on lower limb strength and flexibility; displacement speed; speed, agility, and coordination; and cardiorespiratory endurance in adolescents; (2) to compare outcomes across the three intervention frequencies (1, 3, and 5 days per week); and (3) to examine gender differences in fitness adaptations, we can say that, with respect to lower limb strength, and contrary to expectations, the program did not generate significant improvements in lower limb strength in any of the cohorts. This finding is consistent with previous studies indicating that continuous running-based interventions without an explosive strength component have a limited impact on this capacity [26,27]. However, gender differences increased after the intervention, especially between those who completed the program three and five days a week, with boys increasing their advantage. This could be explained by biological factors (greater muscle mass in boys) and psychosocial factors, such as girls’ lower participation or lower motivation to play sports [28]. From an implementation perspective, these results suggest that supplementing the daily mile with brief resistance exercises (e.g., bodyweight squats during laps) could address this gap without compromising ecological validity [29].

Regarding flexibility, no overall improvements were observed either, although girls who completed the program 5 days a week outperformed boys in this cohort, who experienced significant declines. This suggests that continuous walking or running without specific stretching may not be sufficient to maintain or improve flexibility, especially in boys, in line with recent findings [30,31]. Including dynamic stretching routines after exercise could mitigate this effect.

Displacement speed was the component that showed the greatest improvements, with significant reductions in test time across all cohorts, especially in the intervention group that completed the program 5 days per week. These results support the hypothesis that the daily mile, by combining movements at different intensities (at the participant’s discretion), promotes speed-related neuromuscular adaptations [32,33,34]. However, gender differences persisted, highlighting the importance of incorporating specific strategies to improve girls’ motivation [35,36].

Regarding agility and coordination, a non-significant increase in execution time was observed after the intervention, contrary to expectations. This result could be due to accumulated fatigue or a lack of specificity in the exercises, as agility requires changes in direction, which are not emphasized in the daily mile [37]. Practical solutions could include weekly “obstacle return days” using existing playground markings, balancing specificity with minimal teacher burden [38].

Finally, regarding cardiorespiratory endurance, significant improvements were detected in IG1 and IG3, supporting the effectiveness of the program even at low frequency (1 day/week) [39]. This is consistent with recent studies highlighting the benefits of brief but consistent interventions in sedentary adolescents [39,40]. However, gender differences in C2 suggest that motivational or physiological factors could modulate these adaptations [41,42]. These results may be due to the fact that, although the daily mile is run at one’s own pace, the motivational element may be an important factor to take into account.

In summary, we can state that, contrary to expectations, no clear dose–response relationship was found with respect to intervention frequency. For example, speed improved more in IG3 (5 days/week), but endurance showed similar improvements in IG1 (1 day) and IG3 (3 days). In strength, despite the difference in training frequency, this did not vary significantly between the different cohorts. These results could suggest that other factors, such as intensity or motivation, may be more relevant than weekly training frequency, especially in adolescents with low initial physical fitness [43]. This highlights the need for multi-component school-tailored programs [44], where schools can select frequencies according to their capacity and still achieve significant benefits [45].

Regarding gender, initial differences and disparities were maintained or increased in strength and speed, with boys showing persistent advantages. In cardiorespiratory endurance, boys with higher training frequencies (3 and 5 days per week) maintained their superiority, while girls with a frequency of one day per week improved significantly [46]. Tailoring the intervention through gender-sensitive strategies (e.g., mixed-group activities or self-paced goals) could address these differences [47].

Regarding the study’s limitations, the first worth mentioning is that this intervention may be considered short-term, and therefore, 12 weeks may not be sufficient to assess neuromuscular adaptations. Furthermore, the intensity with which participants performed the urban mile was not exhaustively monitored. Therefore, monitoring heart rate or walking or running speed could have helped to better clarify the dose–response relationship. The use of a convenience sampling method, which limits generalizability and may introduce selection bias. The high dropout rate of the initially selected sample, which may compromise statistical power and internal validity. The absence of a real control group, which prevents establishing causal inferences about the effects of the intervention compared to the absence of intervention. Psychosocial factors, such as motivation, were not assessed, although these could explain gender differences. Using a GPS would have been helpful to determine whether the boys were performing HIIT and the girls were moderately walking.

## 5. Conclusions

This study evaluated the effects of a 12-week Daily Mile program on different components of physical fitness in high school students, analyzing three intervention frequencies (1, 3, and 5 days per week) and gender differences. While all frequencies (1, 3, and 5 days per week) improved cardiorespiratory fitness, the most notable improvements were seen in girls with 1 day per week (IG1) and boys with 3 days per week (IG3). Regarding displacement speed, all groups showed significant improvements, although boys showed greater improvements, especially in IG2 and IG3.

The higher frequency groups (IG2 and IG3) accentuated existing gender disparities in lower limb strength, with boys performing better. Changes in flexibility were minimal overall, although girls in IG3 showed slight improvements, while boys in the same group experienced declines.

These findings suggest that while the daily mile effectively improves aerobic capacity and speed at all frequencies, its implementation needs to be tailored: lower frequencies may be sufficient for girls’ endurance gains, while boys benefit more from higher frequencies. 

## 6. Practical Applications

Based on the results of this study, the following implications for practice emerge: (1) The daily mile is effective for endurance and speed but should be combined with strength training (e.g., jumping, squats) or other types of training to reduce gender gaps. (2) Activity-specific stretches should be performed to maintain flexibility. (3) Since even 1 day/week resulted in improvements in some aspects, such as endurance, the frequency of such programs could be made more flexible, prioritizing adherence over frequency.

## Figures and Tables

**Table 1:** Intervention group characterization.

	Intervention Group 1 (n = 22)	Intervention Group 2 (n = 21)	Intervention Group 3 (n = 25)	p-Value
**Variables**				
**Average age (years)**	14.45 ± 1.14	14.52 ± 1.16	14.40 ± 1.01	0.930 ^b^
**Gender**				0.224 ^a^
Men	15 (68.18%)	13 (61.90%)	19 (76.00%)	
Women	7 (31.82%)	8 (39.10%)	6 (24.00%)	
**Height (m)**	1.62 ± 7.87	1.62 ± 9.15	1.62 ± 6.60	0.977 ^b^
**Weight (kg)**	63.52 ± 13.66	57.94 ± 13.75	54.88 ± 12.44	0.088 ^b^
**BMI (kg/m^2^)**	24.04 ± 3.90	21.84 ± 4.08	20.83 ± 4.01	0.025 ^b^

Note: Quantitative variables are expressed as mean and standard deviation, and qualitative variables as frequencies and percentages. ^a^ Chi-square; ^b^ one-way ANOVA.

**Table 2:** Descriptive data of the variables analyzed (mean, standard deviation) based on gender, time and type of intervention.

		Intervention Group 1		Intervention Group 2		Intervention Group 3	
		Pre	Post	Pre	Post	Pre	Post
Variable		M (SD)	M (SD)	M (SD)	M (SD)	M (SD)	M (SD)
BJ (cm)	Men	151.57 ± 20.87	153.57 ± 16.51	175.83 ± 30.90	178.33 ± 28.23	180.09 ± 27.96	180.90 ± 24.88
	Women	130.18 ± 32.27	126.81 ± 25.91 *	123.16 ± 27.29 **	122.50 ± 22.52 ***	123.75 ± 43.63 *	126.25 ± 35.44 **
	Total	138.50 ± 29.69	137.22 ± 25.90	158.27 ± 38.60	159.72 ± 37.39	165.06 ± 40.39	166.33 ± 36.57
SR (cm)	Men	30.28 ± 4.19	28.42 ± 5.31	32.66 ± 7.36	32.33 ± 9.67	28.57 ± 5.97	25.78 ± 6.99 ^†^
	Women	33.15 ± 8.14	33.84 ± 8.66	35.00 ± 6.61	33.87 ± 10.26	32.33 ± 7.52	32.83 ± 5.38 *
	Total	32.15 ± 7.02	31.95 ± 7.96	33.60 ± 6.99	32.95 ± 9.67	27.70 ± 6.51	27.90 ± 7.21
SAC (s.)	Men	12.03 ± 0.71 ^#^	12.39 ± 0.52	10.89 ± 0.51	11.44 ± 0.95	11.70 ± 0.87	12.40 ± 1.53
	Women	13.22 ± 0.97 *^,$^	13.00 ± 0.94	12.67 ± 1.03 ***	12.64 ± 1.27 *	12.12 ± 0.99	12.35 ± 0.11
	Total	12.80 ± 1.05	12.78 ± 0.85	11.73 ± 1.19	12.00 ± 1.24	11.81 ± 0.89	12.38 ± 1.31
DS (s.)	Men	20.17 ± 1.27	19.53 ± 1.67	18.48 ± 1.53	17.66 ± 1.44 ^†^	19.36 ± 2.07	18.28 ± 1.95 ^†^
	Women	22.63 ± 3.47 *^,$^	21.76 ± 3.24 *^,¥,¢^	21.03 ± 2.61 *	20.20 ± 2.16 *^,¢^	20.18 ± 2.70	19.49 ± 2.54
	Total	21.85 ± 3.14	21.05 ± 2.98	19.45 ± 2.32	18.63 ± 2.12	19.56 ± 2.20	18.57 ± 2.11
CRE (stage)	Men	4.21 ± 1.03	5.28 ± 1.38 ^¢^	5.75 ± 1.74	6.30 ± 1.87	5.69 ± 2.53	6.96 ± 2.97 ^†^
	Women	2.68 ± 1.81	3.72 ± 2.31	3.04 ± 1.92 *	3.45 ± 2.00 *	3.40 ± 1.95	4.40 ± 2.54
	Total	3.27 ± 1.70	4.33 ± 2.11	4.73 ± 2.21	5.23 ± 2.33	5.09 ± 2.56	6.28 ± 3.03

Note: M: mean; SD: standard deviation; BJ: Broad jump; SR: Sit and Reach; SAC: speed, agility and coordination (4 × 10 m); DS: displacement speed (10 × 5 m); CRE: cardiorespiratory endurance; * p < 0.050: difference with men of the same group and period; ** p < 0.010; *** p < 0.001; ^†^ p > 0.050 difference with the boys before; ^¢^ p > 0.050 difference with girls before; ^#^ p < 0.050 IG1 vs. IG3 boys before; ^$^ p < 0.050 IG1 vs. IG5 girls before; ^¥^ p < 0.050 IG1 vs. IG5 girls after.

## Data Availability

The data presented in this study are not available in accordance with Regulation (EU) of the European Parliament and of the Council 2016/679 of 27 April 2016 regarding the protection of natural persons with regard to the processing of personal data and the free circulation of these data (RGPD).

## Abbreviations

The following abbreviations are used in this manuscript:

WHO	World Health Organization
MVPA	moderate-to-vigorous physical activity
IG1	Intervention Group 1
IG 2	Intervention Group 2
IG 3	Intervention Group 3
cm	centimeters
m	meters
kg	kilograms
BJ	Broad jump
SR	Sit and Reach
SAC	speed, agility and coordination
DS	displacement speed
CRE	cardiorespiratory endurance
s	seconds
